# Racial/ethnic differences in the outcomes of patients with metastatic breast cancer: contributions of demographic, socioeconomic, tumor and metastatic characteristics

**DOI:** 10.1007/s10549-018-4956-y

**Published:** 2018-10-06

**Authors:** Jin-Xiao Ren, Yue Gong, Hong Ling, Xin Hu, Zhi-Ming Shao

**Affiliations:** 1 Department of Breast Surgery, Key Laboratory of Breast Cancer in Shanghai, Cancer Institute, Fudan University Shanghai Cancer Center, Fudan University, Shanghai, 200032 China; 2 Department of Oncology, Shanghai Medical College, Fudan University, 270 Dong-An Road, Shanghai, 200032 China; 3 Institutes of Biomedical Science, Fudan University, Shanghai, 200032 China

**Keywords:** Metastatic breast cancer, Racial disparity, Breast cancer prognosis, Age group

## Abstract

**Purpose:**

Population-based estimates of racial disparities in metastatic breast cancer are lacking. We quantified the contributions of demographic, socioeconomic, tumor, and metastatic characteristics to racial differences in metastatic breast cancer and characterized the most disproportional subgroup.

**Methods:**

Patients diagnosed with metastatic breast cancer between 2010 and 2014 were identified using the Surveillance, Epidemiology, and End Results database. A multivariable Cox proportional hazards model was used to adjust each set of variables. The excess relative risk of cancer-specific and all-cause death in non-Hispanic black (NHB) versus non-Hispanic white women diagnosed with metastatic breast cancer was expressed as a percentage and was stratified by the age at diagnosis.

**Results:**

We identified 13,066 female patients. NHB women exhibited substantially higher morbidity and mortality than women of other races/ethnicities. The greatest excess mortality risk for NHB women was observed in the young-onset group (18–49 years; hazard ratio: 1.57), followed by the middle-age group (50–64 years; hazard ratio: 1.42); the trend was not significant among the elderly group. Socioeconomic factors stably explained one-half of the excess risk, whereas the contribution of tumor characteristics obviously decreased with age (18–49 years, 40.7%; 50–64 years, 33.9%), and the metastatic pattern accounted for approximately one-tenth of the excess risk. Additionally, the disproportional death burden of NHB women persisted in less aggressive subgroups.

**Conclusions:**

By providing a comprehensive assessment of racial differences in the incidence and outcomes of patients with metastatic breast cancer, we urge the implementation of targeted preventive efforts in both the public health and clinical arenas.

**Electronic supplementary material:**

The online version of this article (10.1007/s10549-018-4956-y) contains supplementary material, which is available to authorized users.

## Introduction

The presence of distant metastases at initial diagnosis represents an important cause of morbidity and mortality among all patients with breast neoplasms, and the burden on the population is unequal. Metastatic breast cancer represents 9% of diagnoses among non-Hispanic black (NHB) women compared with 5–6% of diagnoses in other racial/ethnic groups. Regarding the survival gains in patients with distant-stage disease from 1975 to 2013, the 5-year cause-specific survival of non-Hispanic white (NHW) women (19–37%) was higher than that of other racial/ethnic groups, particularly NHB women (16–26%) [1, 2]. Racial disparities in breast cancer have been well documented but estimates of racial variances in metastatic breast cancer are lacking.

Previous research on racial differences in all-stage or early-stage breast cancer has attributed the disproportionate incidences of these diseases to characteristics at diagnosis, such as age, socioeconomic status (SES), and tumor biology [3–7]. A recent study examined the black-white disparity in non-elderly women diagnosed with stage I to III breast cancer from 2004 to 2013 in the National Cancer Data Base, finding that differences in insurance coverage accounted for one-third of the survival disparity, whereas differences in tumor characteristics accounted for one-fifth of the disparity [8]. According to another recent study on patients with all-stage breast cancer conducted using only California Cancer Registry data, the stage at diagnosis accounted for 24% of the differences in cancer-specific survival, whereas hormone receptor (HoR) status accounted for 9% and neighborhood SES accounted for 6% [9]. A recent review concluded that racial differences in the stage at diagnosis were not significant but rather gained importance by highlighting differences in tumor biology, patterns of care and other prognostic factors [10]. Here, we estimate stage-specific racial disparities in metastatic breast cancer and examine the contributions of relevant prognostic factors. In addition, the metastatic pattern, which has been reported to display a significantly correlation with the prognosis of metastatic breast cancer, has not yet been investigated in studies using mediating models for racial disparities.

The primary purpose of this study was to quantitatively estimate the contributions of demographic, socioeconomic, tumor and metastatic characteristics to racial differences in metastatic breast cancer using the Surveillance, Epidemiology, and End Results (SEER) database at the population level. We also sought to characterize independent clinical predictors of the variances in survival by race in patients with metastatic breast cancer to improve prevention through suitable clinical strategies and public interventions.

## Methods

### Data source

We obtained population-based data from the SEER 18 registry research database. SEER covers approximately 27.8% of the US population, including 24.9% of Whites, 25.6% of African Americans, 38.4% of Hispanics, 30.6% of American Indians and Alaskan Natives, 50.4% of Asians, and 66.5% of Hawaiian/Pacific Islanders (based on the 2010 Census) [11]. The data reported in this study represent the most recent follow-up (November 2017 Submission) available in the SEER database [12].

### Cohort selection

**Fig. 1:**
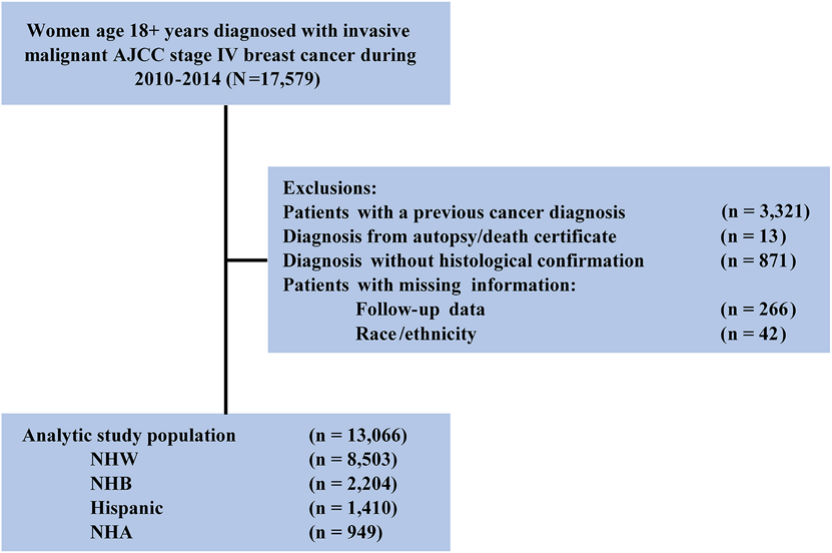
Schematic representation of the patient selection process. *AJCC* American Joint Committee on Cancer, *NHA* non-Hispanic Asian or Pacific Islander/American Indian/Alaskan Native, *NHB* non-Hispanic black, *NHW* non-Hispanic white

We identified 17,579 female adults (aged > 18 years) who were diagnosed with adjusted American Joint Committee on Cancer (AJCC) seventh edition stage IV breast cancer between 2010 and 2014. This time frame was selected because information on distant metastases to specific sites and molecular subtypes at the time of the initial cancer diagnosis was available only for patients who were diagnosed after 2010. We excluded patients with a previous cancer diagnosis (*n* = 3321); patients diagnosed at autopsy or on a death certificate (*n* = 13); patients lacking histological confirmation (*n* = 871); patients with missing follow-up data (*n* = 266); and patients whose race/ethnicity was not available (*n* = 42). Our final cohort included 13,066 women, comprising 74.3% of all 17,579 previously identified patients (Fig. 1).

### Covariate and outcome measures

Our primary outcome of interest was vital status, including follow-up time, breast cancer-specific survival (BCSS), overall survival (OS), and excess relative risk (ERR) of death among different racial groups (versus NHW). The follow-up time was defined as the time between the date of diagnosis and the date of death or the last follow-up visit. BCSS was defined as the interval from the date of diagnosis to the date of death due to breast cancer. OS was defined as the interval from the date of diagnosis to the date of death from any cause.

Race/ethnicity in the SEER database was classified into 5 major groups, including NHW, NHB, non-Hispanic Asian or Pacific Islander (NHAPI), non-Hispanic American Indian/Alaska Native (NHAIAN), and Hispanic (all races). Given the small number of NHAPI and NHAIAN women, we merged the two groups into one. Thus, we classified race/ethnicity into four mutually exclusive groups: (1) NHW, (2) NHB, (3) Hispanic (all races), and (4) Non-Hispanic Asian or Pacific Islander/American Indian/Alaskan Native (NHA).

Neighborhood SES is a county-level, time-dependent composite index developed through principal components analysis of census tract data on education, occupation, employment, household income, poverty, rent and house value. The composite SES score is categorized into quintiles with roughly equal populations, ranging from lowest to highest SES [13, 14].

### Statistical analyses

The incidence and mortality rates were standardized to the 2000 US standard population by age and were expressed per 100,000 persons, as calculated using NCI SEER*Stat software (version 8.3.5) [12, 15].

Kruskal–Wallis tests and Pearson’s *χ*^2^ tests were employed to compare the characteristics of patients stratified by racial/ethnic group for continuous and categorical variables, respectively. Detailed classification information is presented in Table 1. Patients with missing data for a given variable were excluded from the comparative analysis. Univariate and multivariate Cox proportional hazards models were used to identify prognostic factors for OS in the cohort patients. As a non-trivial proportion of the patients died from causes other than breast cancer in the study cohort, Fine and Gray’s competing risk model was employed to compare BCSS [16, 17]. The variables employed for adjustment were modeled as covariates to estimate the contribution of each factor or set of factors to racial disparity. Model one was the unadjusted model. Demographics (age at diagnosis), SES (individual-level insurance and marital status, together with area-level neighborhood SES), tumor characteristics (histological type, grade, tumor size, number of positive regional lymph nodes, and molecular subtype) and metastatic patterns (number and site of distant metastases) were adjusted in models two to five, respectively. Finally, model six adjusted for all variables. Hazard ratios (HRs) and associated 95% confidence intervals (CIs) were estimated from both the BCSS and OS for each model. Next, the ERR of death was calculated by subtracting one from the HR. Finally, the proportion of total ERR explained by each set of variables, as well as by all variables combined, was calculated. Analyses of subgroups stratified according to baseline characteristics were used to evaluate the consistency of racial disparities in survival after adjusting for all other prognostic factors.

**Table 1:** Demographic and clinicopathological characteristics of patients stratified by race/ethnicity

Characteristic	Total	NHW	NHB	Hispanic	NHA	*P* value^a^
	*n* = 13,066 (%)	*n* = 8503 (%)	*n* = 2204 (%)	*n* = 1410 (%)	*n* = 949 (%)	
Age at diagnosis, years						
Median (IQR)	61 (51–71)	63 (54–73)	58 (49–67)	55 (46–65)	57 (48–65)	< 0.001
Insurance status						
Insured	9539 (73.0)	6827 (80.3)	1306 (59.3)	766 (54.3)	640 (67.4)	< 0.001
Medicaid	2636 (20.2)	1209 (14.2)	652 (29.6)	524 (37.2)	251 (26.4)	
Uninsured	891 (6.8)	467 (5.5)	246 (11.2)	120 (8.5)	58 (6.1)	
Marital status						
Married	5552 (42.5)	3896 (45.8)	560 (25.4)	605 (42.9)	491 (51.7)	< 0.001
Not married^b^	6751 (51.7)	4123 (48.5)	1490 (67.6)	735 (52.1)	403 (42.5)	
Unknown	763 (5.8)	484 (5.7)	154 (7.0)	70 (5.0)	55 (5.8)	
Neighborhood SES						
Q1 (low)	2515 (19.2)	1045 (12.3)	877 (39.8)	450 (31.9)	143 (15.1)	< 0.001
Q2	2624 (20.1)	1583 (18.6)	524 (23.8)	332 (23.5)	185 (19.5)	
Q3	2747 (21.0)	1898 (22.3)	354 (16.1)	287 (20.4)	208 (21.9)	
Q4	2596 (19.9)	1954 (23.0)	256 (11.6)	183 (13.0)	203 (21.4)	
Q5 (high)	2419 (18.5)	1918 (22.6)	162 (7.4)	141 (10.0)	198 (20.9)	
Missing	165 (1.3)	105 (1.2)	31 (1.4)	17 (1.2)	12 (1.3)	
Histological type						
IDC	8782 (67.2)	5551 (65.0)	1576 (71.5)	959 (68.0)	696 (73.3)	< 0.001
ILC	1372 (10.5)	992 (11.7)	174 (7.9)	141 (10.0)	65 (6.8)	
Others	2912 (22.3)	1960 (23.1)	454 (20.6)	310 (22.0)	188 (19.8)	
Grade						
I/II	5005 (38.3)	3442 (40.5)	696 (31.6)	516 (36.6)	351 (37.0)	< 0.001
III/UD	5230 (40.0)	3137 (36.9)	1050 (47.6)	625 (44.3)	418 (44.0)	
Unknown	2831 (21.7)	1924 (22.6)	458 (20.8)	269 (19.1)	180 (19.0)	
Tumor size (mm)						
0–20	1907 (14.6)	1342 (15.8)	281 (12.7)	185 (13.1)	99 (10.4)	< 0.001
21–50	4743 (36.3)	3201 (37.6)	713 (32.4)	483 (34.3)	346 (36.5)	
> 50	3903 (29.9)	2307 (27.1)	771 (35.0)	490 (34.8)	335 (35.3)	
Unknown	2513 (19.2)	1653 (19.4)	439 (19.9)	252 (17.9)	169 (17.8)	
Regional lymph node positive						
No	2860 (21.9)	2040 (24.0)	395 (17.9)	268 (19.0)	157 (16.5)	< 0.001
Yes	8803 (67.4)	5474 (64.4)	1624 (73.7)	1009 (71.6)	696 (73.3)	
Unknown	1403 (10.7)	989 (11.6)	185 (8.4)	133 (9.4)	96 (10.1)	
Molecular subtype						
HER2−/HoR+	6866 (52.5)	4668 (54.9)	1004 (45.6)	695 (49.3)	499 (52.6)	< 0.001
HER2+/HoR+	1930 (14.8)	1211 (14.2)	336 (15.2)	233 (16.5)	150 (15.8)	
HER2+/HoR−	1056 (8.1)	623 (7.3)	185 (8.4)	136 (9.6)	112 (11.8)	
HER2−/HoR−	1612 (12.3)	927 (10.9)	416 (18.9)	170 (12.1)	99 (10.4)	
Unknown	1602 (12.3)	1074 (12.6)	263 (11.9)	176 (12.5)	89 (9.4)	
No. of distant metastases						
1	6806 (52.1)	4535 (53.3)	1106 (50.2)	708 (50.2)	457 (48.2)	< 0.001
> 1	5356 (41.0)	3367 (39.6)	974 (44.2)	594 (42.1)	421 (44.4)	
Unknown	904 (6.9)	601 (7.1)	124 (5.6)	108 (7.7)	71 (7.5)	
Bone						
Yes	8515 (65.2)	5720 (67.3)	1315 (59.7)	884 (62.7)	596 (62.8)	< 0.001
No	4245 (32.5)	2576 (30.3)	848 (38.5)	494 (35.0)	327 (34.5)	
Unknown	306 (2.3)	207 (2.4)	41 (1.9)	32 (2.3)	26 (2.7)	
Brain						
Yes	938 (7.2)	578 (6.8)	184 (8.3)	112 (7.9)	64 (6.7)	0.036
No	11,567 (88.5)	7543 (88.7)	1946 (88.3)	1232 (87.4)	846 (89.1)	
Unknown	561 (4.3)	382 (4.5)	74 (3.4)	66 (4.7)	39 (4.1)	
Liver						
Yes	3412 (26.1)	2184 (25.7)	628 (28.5)	338 (24.0)	262 (27.6)	0.030
No	9218 (70.5)	6033 (71.0)	1513 (68.6)	1016 (72.1)	656 (69.1)	
Unknown	436 (3.3)	286 (3.4)	63 (2.9)	56 (4.0)	31 (3.3)	
Lung						
Yes	3891 (29.8)	2388 (28.1)	752 (34.1)	442 (31.3)	309 (32.6)	< 0.001
No	8636 (66.1)	5749 (67.6)	1386 (62.9)	902 (64.0)	599 (63.1)	
Unknown	539 (4.1)	366 (4.3)	66 (3.0)	66 (4.7)	41 (4.3)	
Follow-up duration in months						
Mean (95% CI)	22.6 (22.3–23.0)	23.0 (22.6–23.4)	20.1 (19.4–20.8)	23.9 (23.0-24.9)	23.5 (22.3–24.6)	
Median (IQR)	19.0 (8.0–34.0)	20.0 (7.0–35.0)	17.0 (6.0–30.0)	20.0 (10.0–35.0)	20.0 (8.0–36.0)	
Follow-up status^c^						
Alive	5340 (40.9)	3521 (41.4)	735 (33.3)	636 (45.1)	448 (47.2)	< 0.001
Dead due to breast cancer	6999 (53.6)	4507 (53.0)	1328 (60.3)	706 (50.1)	458 (48.3)	< 0.001
Dead due to other causes	727 (5.6)	475 (5.6)	141 (6.4)	68 (4.8)	43 (4.5)	0.097

*CI* confidence interval, *HER2* human epidermal growth factor receptor-2, *HoR* hormone receptor, *IDC* invasive ductal carcinoma, *ILC* invasive lobular carcinoma, *IQR* interquartile range, *NHA* non-Hispanic Asian or Pacific Islander/American Indian/Alaska Native, *NHB* non-Hispanic black, *NHW* non-Hispanic white, *Q* quintile, *SES* socioeconomic status, *UD* undifferentiated

^a^*P* value was assessed using the Pearson’s *χ*^2^

^b^Including divorced, separated, single (never married), and widowed

^c^As of December 31, 2017

The significance level of the *P* value was set to 0.05 for a two-tailed analysis. Statistical analyses were performed using R version 3.3.3 (R Foundation for Statistical Computing, Vienna, Austria) and SPSS version 22.0 (SPSS, Inc., Chicago, IL).

## Results

### Incidence and mortality rates of metastatic breast cancer among women

**Fig. 2:**
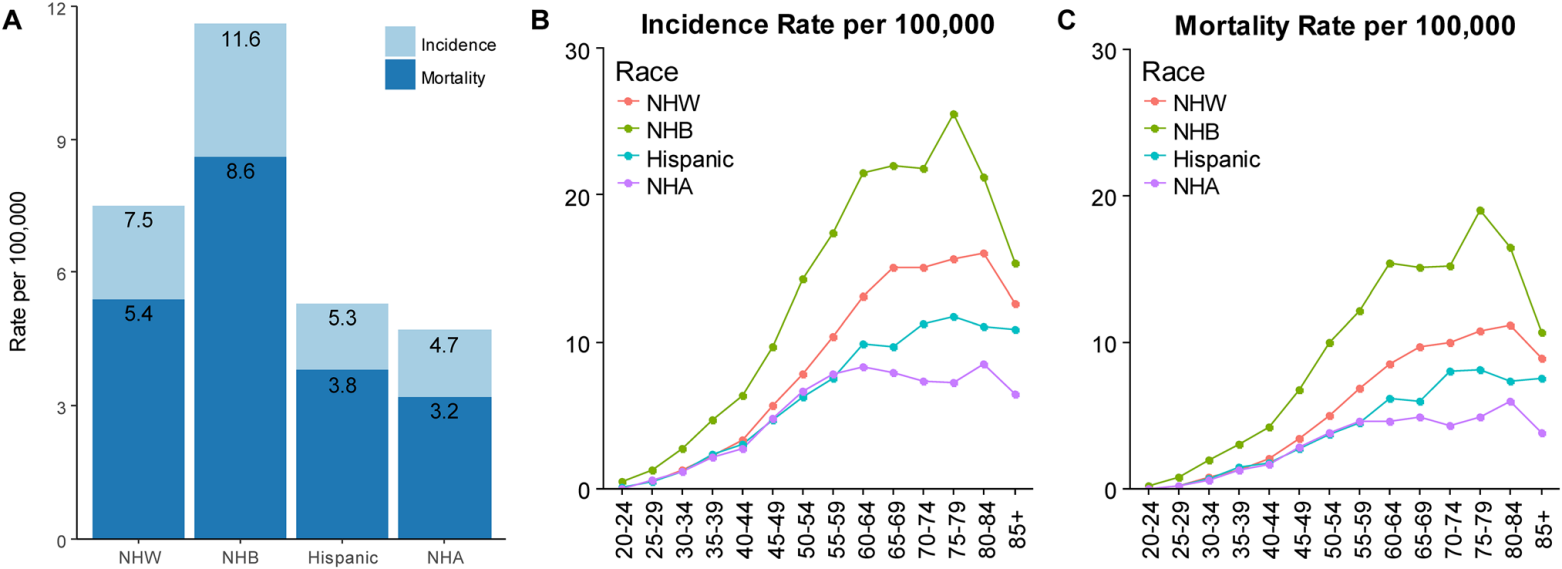
Incidence of metastatic breast cancer and incidence-based mortality rates (2010–2014) and age-specific rates (2010–2014) in female patients stratified by race/ethnicity. The rates were adjusted for age based on the US standard population in 2000. *NHA* non-Hispanic Asian or Pacific Islander/American Indian/Alaskan Native, *NHB* non-Hispanic black, *NHW* non-Hispanic white. *Sources* North American Association of Central Cancer Registries, 2017

The incidence and mortality rates (2010–2014) of metastatic breast cancer among women varied substantially by race/ethnicity in the United States (Fig. 2a). NHB women had exhibited higher incidence and mortality rates than women of other races/ethnicities, whereas NHA women exhibited the lowest incidence and mortality rates. The disparities in the morbidity and mortality of metastatic breast cancer among races/ethnicities remained relatively stable between 2005 and 2014 (the most recent 10 years of data available) (Fig. S1A-B) as well as in trends stratified by HoR status (Fig. S1C-D). The trends in the incidence of HoR + metastatic breast cancer (2005–2014) among the groups stratified by race/ethnicity (Fig. S1C) showed a significant increase among NHW (4.3% per year), NHB (4.8% per year), and NHA women (3.5% per year), but a stable trend among Hispanic women. Racial differences in the incidence and mortality rates of metastatic breast cancer varied according to age (Fig. 2b, c). The incidence rate of distant-stage breast cancer in NHW women was parallel to that of Hispanic or NHA women before the age of 50 years but was much higher thereafter. NHA women displayed lower rates and a better prognosis after the age of 65 years. NHB women were more likely to die from breast cancer at any age.

### Patient characteristics

Of the 13,066 women with metastatic breast cancer at the time of diagnosis, 8503 (65.1%) were NHW; 2204 (16.9%) were NHB; 1410 (10.8%) were Hispanic; and 949 (7.3%) were NHA (Table 1). The median length of follow-up was 19 months (mean, 22.6 months). Overall, patient characteristics were significantly different among the different racial groups. Compared with NHW women, Hispanic women were, on average, 8 years younger at presentation (63 vs. 55 years) and were more likely to have Medicaid insurance (37.2% vs. 14.2%); NHB women were more likely to have poor socioeconomic support (11.2% vs. 5.5% for uninsured; 67.6% vs. 48.5% for unmarried; 39.8% vs. 12.3% for lowest SES neighborhoods); NHB women were also substantially more likely to present with high-grade tumors (47.6% vs. 36.9%) with positivity of regional lymph nodes (73.7% vs. 64.4%) and the human epidermal growth factor receptor-2 (HER2)−/HoR− subtype (18.9% vs. 10.9%). NHW women were the most likely group to be diagnosed with smaller-sized tumors (15.8%), the HER2−/HoR+ subtype (54.9%), a single distant metastasis (53.3%), or bone metastasis (67.3%).

### Differences in the outcomes of groups stratified by race/ethnicity

Univariate and multivariate proportional hazards models for both OS and BCSS were employed to determine prognostic factors for patients with metastatic breast cancer (Tables 2, S1). The death risk was substantially higher in NHB women compared with that in NHW women, and this risk was mitigated in the fully adjusted model (HR for BCSS, 1.24 vs. 1.07; HR for OS, 1.27 vs. 1.12). Hispanic women exhibited a better survival rate in both the univariate and multivariate analyses and the prognosis of NHA women was not significant in the multivariate model. Additionally, after adjusting for all the listed prognostic factors in the multivariate analysis, patients who were diagnosed at an older age with worse socioeconomic support (uninsured, unmarried, and poor SES neighborhoods), advanced tumor characteristics (grade III/UD, tumor size > 50 mm, and HER2−/HoR− subtype), and known specific organ metastases (bone/brain/liver/lung) were at an increased risk of death. Patients with a young onset, a higher SES and the HER2+/HoR + subtype showed a decreased risk of death (*P* < 0.05).

**Table 2:** Univariate and multivariate Fine and Gray model analyses for breast cancer-specific mortality

	No. of patients	No. of deaths	Univariate analysis		Multivariate analysis	
			CIF (95% CI)	*P*	CIF (95% CI)	*P*
Race						
NHW	8503	4507	Reference	–	Reference	–
NHB	2204	1328	1.24 (1.16–1.31)	< 0.001	1.07 (1.00–1.15)	0.047
Hispanic	1410	706	0.92 (0.85–0.99)	0.034	0.91 (0.84–0.99)	0.029
NHA	949	458	0.91 (0.83–0.99)	0.035	0.94 (0.85–1.04)	0.250
Age at diagnosis, years						
1-year increase			1.01 (1.01–1.02)	< 0.001	1.02 (1.01–1.02)	< 0.001
Insurance status						
Insured	9539	4951	Reference	–	Reference	–
Medicaid	2636	1522	1.19 (1.13–1.26)	< 0.001	1.17 (1.10–1.25)	< 0.001
Uninsured	891	526	1.35 (1.23–1.48)	< 0.001	1.30 (1.18–1.44)	< 0.001
Marital status						
Married	5552	2689	Reference	–	Reference	–
Not married^a^	6751	3902	1.35 (1.29–1.42)	< 0.001	1.16 (1.10–1.22)	< 0.001
Neighborhood SES						
Q1 (low)	2515	1473	Reference	–	Reference	–
Q2	2624	1457	0.93 (0.87-1.00)	0.062	0.94 (0.87–1.01)	0.110
Q3	2747	1480	0.88 (0.82–0.95)	< 0.001	0.90 (0.84–0.97)	0.007
Q4	2596	1341	0.80 (0.75–0.87)	< 0.001	0.86 (0.79–0.93)	< 0.001
Q5 (high)	2419	1169	0.73 (0.67–0.78)	< 0.001	0.80 (0.74–0.87)	< 0.001
Histological type						
IDC	8782	4570	Reference	–	Reference	–
ILC	1372	721	0.98 (0.91–1.05)	0.560	1.12 (1.03–1.22)	0.011
Others	2912	1708	1.25 (1.18–1.32)	< 0.001	1.09 (1.02–1.16)	0.007
Grade						
I/II	5005	2300	Reference	–	Reference	–
III/UD	5230	3062	1.46 (1.38–1.53)	< 0.001	1.35 (1.27–1.43)	< 0.001
Tumor size(mm)						
0–20	1907	925	Reference	–	Reference	–
21–50	4743	2303	1.00 (0.93–1.08)	0.930	1.03 (0.95–1.11)	0.520
> 50	3903	2194	1.27 (1.18–1.37)	< 0.001	1.20 (1.11–1.30)	< 0.001
Regional lymph nodes positive						
No	2860	1507	Reference	–	Reference	–
Yes	8803	4573	0.94 (0.89-1.00)	0.035	0.96 (0.90–1.02)	0.170
Molecular subtype						
HER2−/HoR+	6866	3389	Reference	–	Reference	–
HER2+/HoR+	1930	828	0.84 (0.78–0.90)	< 0.001	0.77 (0.71–0.83)	< 0.001
HER2+/HoR−	1056	501	1.02 (0.93–1.12)	0.660	0.90 (0.81–0.99)	0.028
HER2−/HoR−	1612	1224	2.27 (2.12–2.42)	< 0.001	2.04 (1.89–2.20)	< 0.001
No. of distant metastases						
1	6808	3224	Reference	–	Reference	–
> 1	5356	3186	1.47 (1.40–1.55)	< 0.001	1.09 (1.03–1.15)	0.004
Bone						
Yes	8515	4583	1.02 (0.97–1.08)	0.380	1.23 (1.16–1.30)	< 0.001
No	4245	2217	Reference	–	Reference	–
Brain						
Yes	938	6567	2.23 (2.06–2.42)	< 0.001	1.96 (1.79–2.13)	< 0.001
No	11,567	5926	Reference	–	Reference	–
Liver						
Yes	3412	2209	1.71 (1.62–1.80)	< 0.001	1.77 (1.67–1.88)	< 0.001
No	9218	4513	Reference	–	Reference	–
Lung						
Yes	3891	2370	1.47 (1.39–1.54)	< 0.001	1.24 (1.17–1.31)	< 0.001
No	8636	4277	Reference	–	Reference	–

*CI* confidence interval, *HER2* human epidermal growth factor receptor-2, *HoR* hormone receptor, *HR* hazard ratio, *IDC* invasive ductal carcinoma, *ILC* invasive lobular carcinoma, *NHA* non-Hispanic Asian or Pacific Islander/American Indian/Alaska Native, *NHB* non-Hispanic black, *NHW* non-Hispanic white, *Q* quintile, *SES* socioeconomic status, *UD* undifferentiated

^a^Including divorced, separated, single (never married), and widowed

HRs for cancer-specific death between NHB and NHW women were attenuated after adjusting for socioeconomic, tumor or metastatic characteristics, but these HRs were increased in age-adjusted models. These factors together accounted for 69.5% of the total excess risk of death in NHB women compared with that in NHW women. In the respective adjusted models, the estimated proportion of excess risk attributed to socioeconomic factors was 66.5%, followed by tumor characteristics at 41.5%, and the metastatic pattern at 14.8%. Conversely, with the imbalance of the age distribution, the racial differences were aggravated by 40.3% (Table 3).

**Table 3:** HRs for death resulting from breast cancer in NHB and NHW patients

Age	Race	No. of patients	No. of deaths	Model one^a^	Model two^b^	Model three^c^	Model four^d^	Model five^e^	Model six^f^
				HR (95% CI)	HR (95% CI)	HR (95% CI)	HR (95% CI)	HR (95% CI)	HR (95% CI)
Total	NHW (reference)	8503	4507	1.00	1.00	1.00	1.00	1.00	1.00
	NHB v NHW	2204	1328	1.24 (1.16–1.31)	1.33 (1.25–1.42)	1.08 (1.01–1.15)	1.14 (1.07–1.21)	1.20 (1.13–1.28)	1.07 (1.00–1.15)
	ERR, %			23.6	33.1	7.9	13.8	20.1	7.2
	Explainable ERR, %				− 40.3	66.5	41.5	14.8	69.5
18–49	NHW (reference)	1384	575	1.00	1.00	1.00	1.00	1.00	1.00
	NHB v NHW	589	320	1.57 (1.37–1.80)	1.60 (1.39–1.81)	1.28 (1.10–1.49)	1.34 (1.15–1.55)	1.48 (1.28–1.71)	1.13 (0.96–1.34)
	ERR, %			57.0	59.5	27.7	33.8	48.0	13.4
	Explainable ERR, %				− 4.3	51.4	40.7	15.8	76.5
50–64	NHW (reference)	3246	1685	1.00	1.00	1.00	1.00	1.00	1.00
	NHB v NHW	981	617	1.42 (1.30–1.56)	1.44 (1.31–1.57)	1.20 (1.08–1.32)	1.28 (1.16–1.40)	1.41 (1.29–1.55)	1.11 (1.00–1.23)
	ERR, %			42.2	43.5	19.6	27.9	40.9	10.8
	Explainable ERR, %				− 3.1	53.6	33.9	3.1	74.4
65+	NHW (reference)	3873	2247	1.00	1.00	1.00	1.00	1.00	1.00
	NHB v NHW	634	391	1.10 (0.99–1.22)	1.12 (1.01–1.25)	1.04 (0.93–1.17)	1.04 (0.93–1.16)	1.09 (0.98–1.21)	1.01 (0.90–1.13)

*CI* confidence interval, *ERR* excess relative risk, *HR* hazard ratio, *NHB* non-Hispanic black, *NHW* non-Hispanic white

^a^Model one: race

^b^Model two: race, plus age at diagnosis

^c^Model three: race, plus socioeconomic factors (insurance type, marital status and neighborhood socioeconomic status)

^d^Model four: race, plus tumor characteristics (histological type, grade, tumor size, regional lymph nodes, and molecular subtype)

^e^Model five: race, plus metastatic pattern (number and site of distant metastases)

^f^Model six: race, plus age at diagnosis, socioeconomic factors, tumor characteristics, and metastatic pattern

Table 3 further lists HRs for death resulting from breast cancer, in NHB and NHW women stratified by age. Among patients who were diagnosed with metastatic breast cancer at a younger age (18–49 years), NHB women were 57.0% more likely to die than NHW women (HR 1.57; 95% CI 1.37–1.80) and the excess risk was decreased to 13.4% (HR 1.13; 95% CI 0.96–1.34) after multivariate adjustment. These factors together accounted for 76.5% of the total excess risk; in the respective adjusted models, the excess risk attributed to socioeconomic factors was 51.4%, followed by tumor characteristics at 40.7%, and the metastatic pattern at 15.8%. Among middle-aged patients (50–64 years), NHB women were 42.2% more likely to die than NHW women (HR 1.42; 95% CI 1.30–1.56), and the excess risk decreased to 10.8% (HR 1.11; 95% CI 1.00–1.23) in the fully adjusted model. These factors together accounted for 74.4% of the total excess risk; the excess risk mediated by socioeconomic factors was 53.6%, followed by tumor characteristics at 33.9%, and the metastatic pattern at 3.1%. Among the elderly group (aged 65 + years), no racial difference in breast cancer-specific survival was observed (HR 1.10; 95% CI 0.99–1.22). Table S2 lists the HRs for BCSS in NHW and NHB women with narrowed age categories, excluding the 18–34 age group, due to an insufficient number of events and lack of statistical significance. Consistent with the above analysis, the excess mortality risk of NHB women was increased significantly in the younger age group (aged < 45 years) and non-significantly among the elderly group (aged 65 + years). The results were similar for total mortality (Table S3).

### Subgroup analysis of NHW and NHB women

**Fig. 3:**
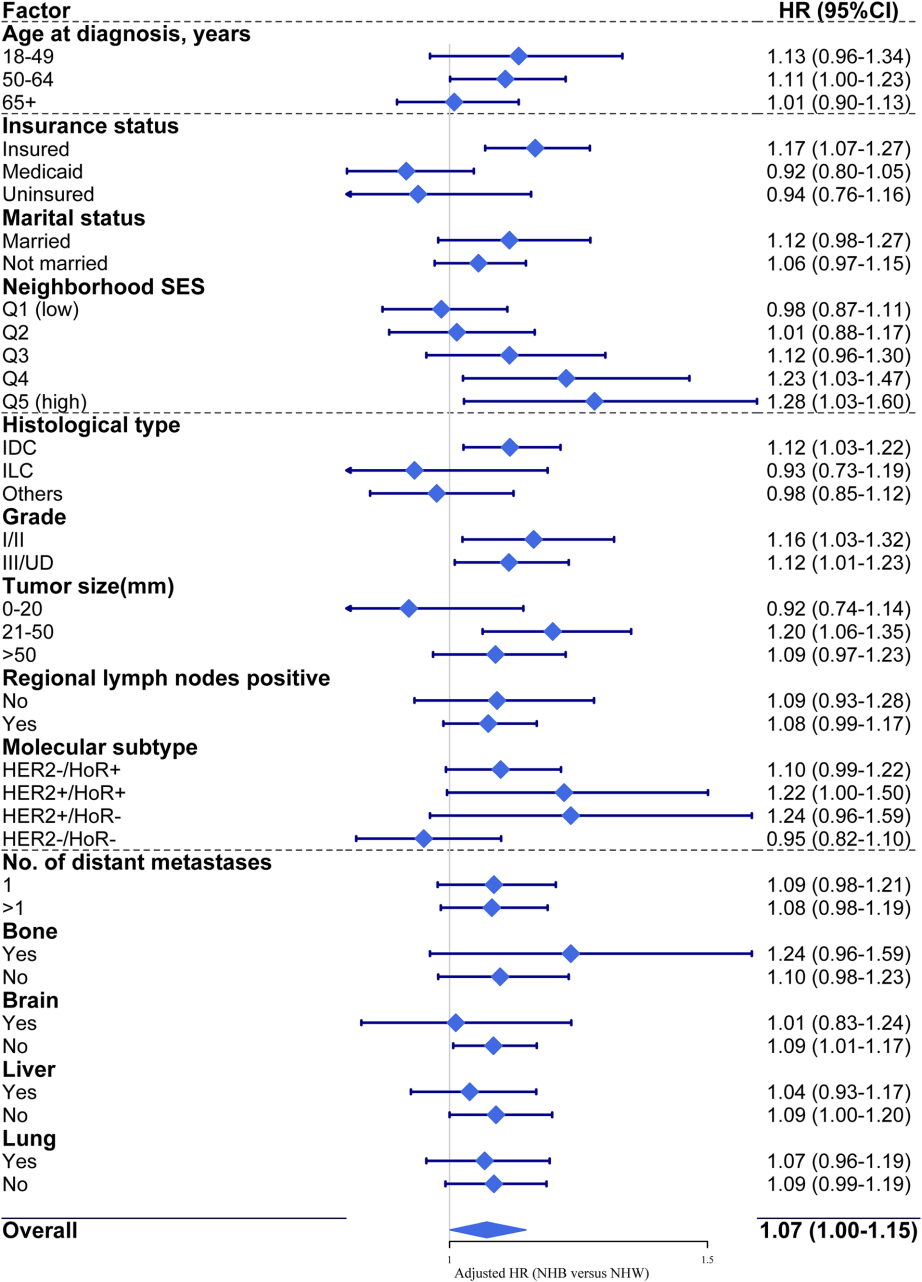
Forest plot showing the results of the multivariate proportional hazards model analysis of breast cancer-specific mortality in NHB and NHW women stratified by subgroup after adjustment for all other prognostic factors listed. The diamond denotes the HR of each subgroup. An HR > 1.0 indicates a higher risk of breast cancer-specific mortality in NHB women. *CI* confidence interval, *HER2* human epidermal growth factor receptor-2, *HoR* hormone receptor, *HR* hazard ratio, *IDC* invasive ductal carcinoma, *ILC* invasive lobular carcinoma, *NHB* non-Hispanic black, *NHW* non-Hispanic white, *Q* quintile, *SES* socioeconomic status, *UD* undifferentiated

After adjustment for all listed prognostic factors in the model of BCSS, prognostic differences in NHB and NHW women persisted in less aggressive subgroups with higher-SES individuals and neighborhoods, tumors at the T2 stage (21–50 mm) with the ductal histological type, and a lack of existing brain metastases (Fig. 3).

## Discussion

Population-based estimates of the racial disparities in metastatic breast cancer are lacking. By providing a comprehensive assessment of racial differences in the incidence and outcomes of patients with de novo metastatic breast cancer, this retrospective study confirmed and extended the higher risk of NHB women coupled with a large population, a precise definition of subtype and detailed information on metastatic sites. The two dominant contributors to these racial differences were socioeconomic factors and tumor characteristics, the latter of which further explained the strengthened racial disparities in survival among the younger group. Furthermore, white-black survival disparities persisted in less aggressive subgroups, underscoring the importance of implementing more intensive strategies for detecting and treating metastases in non-elderly NHB women with better SES, tumors at the T2 stage with the ductal histological type, and a lack of existing brain metastases.

According to national epidemiologic data [1, 2, 18], the overall incidence of breast cancer (2005–2014) is higher in NHW women in the US; however, for metastatic breast cancer, NHB women showed a higher incidence in the present study. A decreasing trend in the mortality rate (2005–2014) for all-stage breast cancer but a stable trend for metastatic breast cancer were observed. Moreover, NHB women showed significantly higher morbidity and mortality of metastatic breast cancer at any age.

However, the extent of racial variances changed when the data were stratified by age. We found more substantial differences in both BCSS and OS in younger patients with metastatic breast cancer, whereas an excess risk of death was not observed among elderly NHB women. A study from Vaz et al. [6] focused on elderly patients with de novo metastatic breast cancer who were diagnosed between 1998 and 2009 supported a non-significant white-black difference in OS. Regarding racial disparities in survival among patients with all-stage breast cancer, Sighoko et al. [3] reported similar results for cohorts stratified by age in ten US cities with large African American populations; nevertheless, baseline characteristics other than age were not provided due to a lack of information. Taken together, these findings highlight the importance of age-based guidelines for breast cancer screening and the early detection of distant metastases and that these guidelines should consider race, particularly for non-elderly NHB women.

Differences in socioeconomic support have previously been cited as reasons for overall racial differences in breast cancer prognoses [7, 13, 19–21]. Low-SES individuals and neighborhoods at diagnosis were consistently associated with worse survival in NHB patients than in NHW patients with breast cancer [22–24]. We extended these conclusions to metastatic breast cancer. A low SES accounted for approximately one-half of the excess survival risk in NHB women compared with that in NHW women (BCSS: total, 66.5%; 18–49 years, 51.4%; 50–64 years, 53.6%; 65 + years, NA; OS: total, 68.9%; 18–49 years, 48.1%; 50–64 years, 50.4%; 65 + years, NA). For the 65 + age group, the racial disparity in risk was not significant, which could be partially explained by relatively uniform health care coverage through Medicare in this age group compared with other age cohorts. Therefore, for non-elderly patients with no comparable insurance coverage, equal access to care reduces racial/ethnic disparities in prognosis.

Differences in tumor biology have also been consistently cited as contributors to racial/ethnic disparities in breast cancer mortality [5, 6, 9, 10, 25–33]. In the present study, this factor explained approximately two-fifths (41.5%) of the total excess cancer-specific risk. This estimated percentage is larger than that found in previous studies (Warner et al. [34], 23.8%; Jemal et al. [8], 25.8%). However, direct comparison is limited by the differences in the analyzed populations (stage IV vs. stages I to III). Advanced tumor characteristics play a more important role in younger patients with metastatic breast cancer, as mentioned above (BCSS:18–49 years, 40.7%; 50–64 years, 33.9%; 65 + years, NA; OS: 18–49 years, 35.1%; 50–64 years, 24.9%; 65 + years, NA). These results reflect a higher percentage of patients diagnosed at a younger age (< 50 years) among NHB women than among NHW women (26.7% vs. 16.3%), together with an even more disproportionate number of patients with the HER2−/HoR− subtype (20.0% vs. 12.6%), larger tumor size (> 50 mm, 36.5 vs. 26.8), regional lymph node infiltration (79.3% vs. 72.3%), and other aggressive characteristics associated with a poorer survival among young NHB women (Table S4). A similar pattern was observed among patients diagnosed at a median age (50–64 years), who presented smaller disparities, but not among patients aged 65 +. BRCA1/2 gene mutations, particularly the rapidly progressive and highly malignant HER2−/HoR− subtype, are reported to closely correlate with the aggressive young-onset phenotype in NHB women [35–38]. With advances in breast cancer genomics, more comprehensive studies are expected to clarify potential genome-based differences.

Differences in the metastatic pattern explained approximately one-tenth of the excess risk (BCSS: 14.8%; OS: 12.0%), which was narrowed but not eradicated after stratification by age (18–49 years, 15.8%; 50–64 years, 3.1%; 65 + years, NA). The higher proportion of bone-only metastases in NHW women accounted for the substantially better survival [39–42]. In comparison, NHB women exhibited more brain metastases, which were potentially correlated with the higher percentage of the HoR-negative subtype [28, 43].

NHB women presented an absolute disparity in the rate of suffering more aggressive tumors, and a relative disparity in white-black survival also persisted in less aggressive subgroups. Similar results were observed based on SES among all-stage breast cancer and other solid malignancies [9, 21–23]. We extended the conclusion to more comprehensive prognostic factors including demographic, socioeconomic, tumor and metastatic characteristics. Insured NHB patients from high-SES neighborhoods, exhibiting tumors at the T2 stage with the ductal histological type, and without existing brain metastases suffered poor survival compared with their NHW counterparts, suggesting that NHW patients obtained greater benefits from improved access to care. Some possible explanations for this disparity include differences in the accessibility and utilization of and compliance with systemic treatments such as endocrine therapy, HER2-targeted therapy or chemotherapy due to patient-level social, cultural, linguistic, or even financial barriers [6, 44]. Large population-based studies involving more detailed explorations of how treatment patterns affect outcomes among races/ethnicities are warranted in the future. Another potential explanation includes delayed detection resulting from asymptomatic distant metastases among NHB women with seemingly better prognostic factors. Brain MRI for early detection of brain metastases is recommended by Martin et al. [43] for patients with the HER2+/HoR- and HER2-/HoR- subtypes, which comprise more than one-quarter of NHB patients. Efforts to reduce lagging survival among NHB women with metastatic breast cancer are needed in both less and more aggressive groups.

For Hispanics and NHAs, relatively better survival was observed in our study, together with a younger onset and lower SES. These findings warrant additional attention to the civil awareness of breast cancer, disparities in access to care and potential genetic susceptibility. Both the Hispanic and NHA groups are significantly heterogenetic racial/ethnic groups, comprising subgroups that differ in country of origin, primary language, and geographic location. Thus, the survival of Hispanic or NHA patients with metastatic breast cancer varies with the distribution of prognostic factors in each study population [45–48]. Further comparisons among specific subgroups were limited by the small sample size of the available data and warrant future studies.

## Limitations

Our study has several limitations. First, although the NCI SEER database is the largest cancer database in the United States, it still does not cover 100% of the US population, with different percentages of coverage being observed according to race/ethnicity. Nevertheless, the heterogeneity of the population included in this database, coupled with its large sample size, supports the validity of our findings. Additionally, the results presented here are specific to the population distribution in the United States, and caution should be taken in generalization to other geographic regions/countries. Second, information about disease recurrence or subsequent sites involved is not available in the SEER database; thus, we were only able to include de novo metastases rather than relapsed metastatic cases. Future investigations using alternative data sources to include relapsed metastases are warranted. Third, because NCCN guidelines do not recommend routine systemic staging for patients with asymptomatic early-stage breast cancer, underestimation of the incidence of de novo metastatic breast cancer could exist. Fourth, the age-adjusted mortality rates calculated in the present study were not directly based on mortality data collected by the National Center for Health Statistics (1969–2015) because partitioning of mortality by associated variables is permitted only in the Incidence-based Mortality database (2000–2014). Fifth, other potential socioeconomic factors, such as occupation, education, income, and tax, were not examined because patient-level information is not available in the SEER database. Finally, we cannot comment on comorbidities, body mass indexes, family histories, genomic mutations, treatment patterns or toxicities due to a lack of such information.

## Conclusions

Despite all these limitations, our study provides insights into the racial variations in the incidence and outcomes of de novo metastatic breast cancer using recent data obtained from 13,066 women across the United States. As an explanation for the distribution of excess risk for different factors computed in the age-stratified groups, we identified lower survival of non-elderly NHB women than of NHW women, urging the implementation of targeted preventive efforts in public health, policy, and clinical arenas. Furthermore, clinicians should focus more on non-elderly NHB patients in less aggressive subgroups who are more likely to suffer distant metastases than their NHW counterparts. We urge multidimensional verification of and potential intervention in survival disparities in the clinical field.

## Electronic supplementary material

Below is the link to the electronic supplementary material.

Supplementary material 1 (PDF 259 KB)
