# Supplementary material for: Racial/ethnic differences in the outcomes of patients with metastatic breast cancer: contributions of demographic, socioeconomic, tumor and metastatic characteristics
Source: Breast Cancer Res Treat. 2018 Oct 6;173(1):225–37. doi: 10.1007/s10549-018-4956-y (PMC6394580; doi:10.1007/s10549-018-4956-y)
Supplement: Supplementary file 1 — Supplementary material 1 (PDF 259 KB) [file 10549_2018_4956_MOESM1_ESM.pdf]

SUPPLEMENTARY INFORMATION

Racial/ethnic differences in the outcomes of patients with metastatic breast cancer: contributions of demographic, socioeconomic, tumor and metastatic characteristics

Jin-Xiao Ren[1,2†], Yue Gong[1,2†], Hong Ling[1], Xin Hu[1*], Zhi-Ming, Shao[1,2,3*]

[1]Department of Breast Surgery, Key Laboratory of Breast Cancer in Shanghai, Fudan University Shanghai Cancer Center, Fudan University, Shanghai, 200032, China

[2]Department of Oncology, Shanghai Medical College, Fudan University, Shanghai, 200032, China

[3]Institutes of Biomedical Science, Fudan University, Shanghai, 200032, China

†Both authors contributed equally and share co-first authorship.

*Corresponding authors: Xin Hu, e-mail: xinhu@fudan.edu.com; Zhi-Ming Shao, email: zhi_ming_shao@163.com

- 1 Supplementary Figure
- 4 Supplementary Tables

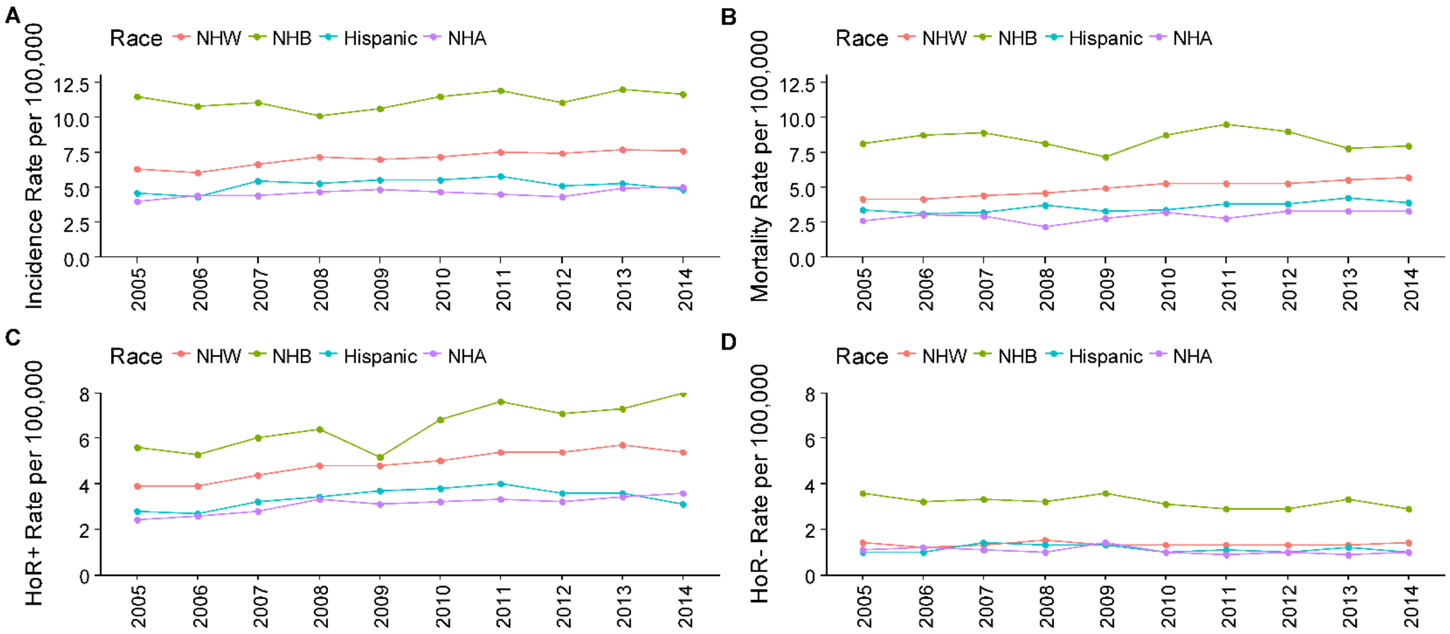

Fig. S1. Trends in the incidence of metastatic breast cancer incidence and incidence-based mortality rates (2005-2014) in female patients in the United States stratified by race/ethnicity. A. Trends in incidence rates. B. Trends in incidence-based mortality rates. C. Trends in the incidence of HoR-positive cases. D. Trends in the incidence of HoR-negative cases. Rates are adjusted for age based on the US standard population in 2000. HoR, hormone receptor; NHA, non-Hispanic Asian or Pacific Islander/American Indian/Alaskan Native; NHB, non-Hispanic black; NHW, non-Hispanic white. Sources: Surveillance, Epidemiology, and End Results program.

Table S1. Univariate and multivariate Cox proportional hazards model analysis for overall mortality

	No. of Patients	No. of Deaths	Univariate analysis		Multivariate analysis	
			HR (95% CI)	P	HR (95% CI)	P
Race						
NHW	8503	4982	Reference	—	Reference	—
NHB	2204	1469	1.27 (1.20-1.35)	<0.001	1.12 (1.05-1.19)	<0.001
Hispanic	1410	774	0.90 (0.84-0.97)	0.008	0.91 (0.84-0.98)	0.015
NHA	949	501	0.88 (0.81-0.97)	0.008	0.95 (0.87-1.04)	0.291
Age at diagnosis, years						
One-year increase			1.02 (1.02-1.02)	<0.001	1.02 (1.02-1.02)	<0.001
Insurance status						
Insured	9539	5486	Reference	—	Reference	—
Medicaid	2636	1677	1.20 (1.20-1.20)	<0.001	1.20 (1.13-1.27)	<0.001
Uninsured	891	563	1.32 (1.32-1.32)	<0.001	1.31 (1.19-1.43)	<0.001
Marital status						
Married	5552	2918	Reference	—	Reference	—
Not married	6751	4349	1.45 (1.45-1.45)	<0.001	1.20 (1.14-1.26)	<0.001
Neighborhood SES						
Q1 (low)	2515	1637	Reference	—	Reference	—
Q2	2624	1613	0.92 (0.86-0.98)	0.014	0.93 (0.86-0.99)	0.033
Q3	2747	1637	0.86 (0.86-0.86)	<0.001	0.87 (0.81-0.94)	<0.001
Q4	2596	1471	0.76 (0.76-0.76)	<0.001	0.81 (0.75-0.88)	<0.001
Q5 (high)	2419	1278	0.68 (0.68-0.68)	<0.001	0.76 (0.70-0.82)	<0.001
Histological type						
IDC	8782	5030	Reference	—	Reference	—
ILC	1372	798	0.98 (0.91-1.06)	0.640	1.09 (1.00-1.18)	0.042
Others	2912	1898	1.30 (1.30-1.30)	<0.001	1.11 (1.05-1.18)	<0.001
Grade						
I/II	5005	2575	Reference	—	Reference	—
III/UD	5230	3323	1.44 (1.44-1.44)	<0.001	1.35 (1.28-1.43)	<0.001
Tumor size(mm)						
0-20	1907	1043	Reference	—	Reference	—
21-50	4743	2567	0.99 (0.92-1.06)	0.690	1.02 (0.95-1.09)	0.648
>50	3903	2390	1.23 (1.23-1.23)	<0.001	1.17 (1.09-1.26)	<0.001
Regional lymph nodes positive						
No	2860	1692	Reference	—	Reference	—
Yes	8803	4993	0.89 (0.89-0.89)	<0.001	0.93 (0.88-0.98)	0.011
Molecular subtype						
HER2-/HoR+	6866	3794	Reference	—	Reference	—
HER2+/HoR+	1930	900	0.79 (0.79-0.79)	<0.001	0.74 (0.69-0.80)	<0.001
HER2+/HoR-	1056	549	1.00 (0.91-1.09)	0.990	0.90 (0.82-0.99)	0.028
HER2-/HoR-	1612	1314	2.34 (2.34-2.34)	<0.001	2.13 (1.98-2.28)	<0.001
No. of distant metastases						
1	6808	3607	Reference	—	Reference	—
>1	5356	3474	1.46 (1.46-1.46)	<0.001	1.09 (1.03-1.15)	0.002
Bone						
Yes	8515	5016	0.98 (0.94-1.03)	0.520	1.18 (1.12-1.25)	<0.001
No	4245	2486	Reference	—	Reference	—
Brain						
Yes	938	765	2.23 (2.23-2.23)	<0.001	1.97 (1.82-2.13)	<0.001
No	11567	6567	Reference	—	Reference	—
Liver						
Yes	3412	2353	1.65 (1.65-1.65)	<0.001	1.78 (1.69-1.88)	<0.001
No	9218	5060	Reference	—	Reference	—
Lung						
Yes	3891	2608	1.51 (1.51-1.51)	<0.001	1.26 (1.20-1.33)	<0.001
No	8636	4732	Reference	—	Reference	—

Abbreviations: CI, confidence interval; HER2, human epidermal growth factor receptor-2; HoR, hormone receptor; HR, hazard ratio; IDC, invasive ductal carcinoma; ILC: invasive lobular carcinoma; NHA, non-Hispanic Asian or Pacific Islander/American Indian/Alaska Native; NHB, non-Hispanic black; NHW, non-Hispanic white; Q, quintile; SES, socioeconomic status; UD, undifferentiated.

a. Including divorced, separated, single (never married), and widowed.

Table S2. HRs for death resulting from breast cancer in NHB and NHW patients

Age	Race	No. of Patients	No. of Deaths	Model one[a] HR (95% CI)	Model two[b] HR (95% CI)	Model three[c] HR (95% CI)	Model four[d] HR (95% CI)	Model five[e] HR (95% CI)	Model six[f] HR (95% CI)
Total	NHW (reference)	8503	4507	1.00	1.00	1.00	1.00	1.00	1.00
	NHB v NHW	2204	1328	1.24 (1.16-1.31)	1.33 (1.25-1.42)	1.08 (1.01-1.15)	1.14 (1.07-1.21)	1.20 (1.13-1.28)	1.07 (1.00-1.15)
	ERR, %			23.6	33.1	7.9	13.8	20.1	7.2
	Explainable ERR, %				-40.3	66.5	41.5	14.8	69.5
18-34	NHW (reference)	188	68	1.00	1.00	1.00	1.00	1.00	1.00
	NHB v NHW	111	51	1.50 (1.04-2.15)	1.50 (1.04-2.15)	1.26 (0.83-1.90)	1.25 (0.84-1.87)	1.33 (0.90-1.96)	1.00 (0.63-1.58)
35-44	NHW (reference)	571	229	1.00	1.00	1.00	1.00	1.00	1.00
	NHB v NHW	261	144	1.67 (1.36-2.07)	1.68 (1.36-2.07)	1.40 (1.12-1.76)	1.43 (1.15-0.78)	1.64 (1.32-2.05)	1.36 (1.07-1.74)
	ERR, %			67.0	67.6	40.3	42.6	64.4	36.1
	Explainable ERR, %				-0.8	39.9	36.4	3.9	46.1
45-54	NHW (reference)	1525	711	1.00	1.00	1.00	1.00	1.00	1.00
	NHB v NHW	537	316	1.49 (1.31-1.70)	1.49 (1.31-1.70)	1.18 (1.02-1.37)	1.29 (1.12-1.48)	1.43 (1.24-1.64)	1.02 (0.88-1.19)
	ERR, %			49.0	49.3	18.2	28.7	42.5	2.3
	Explainable ERR, %				-0.6	62.9	41.4	13.3	95.3
55-64	NHW (reference)	2346	1252	1.00	1.00	1.00	1.00	1.00	1.00
	NHB v NHW	661	426	1.43 (1.28-1.59)	1.43 (1.28-1.59)	1.22 (1.09-1.37)	1.30 (1.17-1.45)	1.42 (1.28-1.59)	1.15 (1.02-1.29)
	ERR, %			42.5	42.6	22.4	30.1	42.2	14.5
	Explainable ERR, %				-0.2	47.3	29.2	0.7	65.9
65-74	NHW (reference)	1974	1082	1.00	1.00	1.00	1.00	1.00	1.00
	NHB v NHW	360	205	1.06 (0.92-1.23)	1.06 (0.91-1.23)	1.02 (0.87-1.19)	0.99 (0.85-1.15)	1.09 (0.93-1.26)	0.97 (0.82-1.13)
75+	NHW (reference)	1899	1165	1.00	1.00	1.00	1.00	1.00	1.00
	NHB v NHW	274	186	1.17 (1.00-1.36)	1.19 (1.01-1.38)	1.12 (0.95-1.31)	1.12 (0.96-1.30)	1.14 (0.98-1.33)	1.06 (0.89-1.26)

Abbreviations: CI, confidence interval; ERR, excess relative risk; HR, hazard ratio; NHB, non-Hispanic black; NHW, non-Hispanic white.

a. Model one: race.

b. Model two: race, plus age at diagnosis.

c. Model three: race, plus socioeconomic factors (insurance type, marital status and neighborhood socioeconomic status).

d. Model four: race, plus tumor characteristics (histological type, grade, tumor size, regional lymph nodes, and molecular subtype).

e. Model five: race, plus metastatic pattern (number and site of distant metastases).

f. Model six: race, plus age at diagnosis, socioeconomic factors, tumor characteristics, and metastatic pattern.

Table S3. HRs for death resulting from any cause in NHB and NHW patients

Age	Race	No. of Patients	No. of Deaths	Model one[a]	Model two[b]	Model three[c]	Model four[d]	Model five[e]	Model six[f]
				HR (95% CI)	HR (95% CI)	HR (95% CI)	HR (95% CI)	HR (95% CI)	HR (95% CI)
Total	NHW (reference)	8503	4982	1.00	1.00	1.00	1.00	1.00	1.00
	NHB v NHW	2204	1469	1.27 (1.20-1.35)	1.41 (1.33-1.50)	1.08 (1.02-1.15)	1.18 (1.12-1.26)	1.24 (1.17-1.31)	1.12 (1.05-1.19)
	ERR, %			27.2	41.1	8.4	18.5	24.9	11.9
	Explainable ERR, %				-51.3	68.9	32.0	12.0	56.3
18-49	NHW (reference)	1384	605	1.00	1.00	1.00	1.00	1.00	1.00
	NHB v NHW	589	345	1.67 (1.46-1.91)	1.70 (1.49-1.93)	1.35 (1.17-1.56)	1.44 (1.25-1.65)	1.60 (1.40-1.83)	1.25 (1.08-1.45)
	ERR, %			67.2	70.4	34.9	43.6	60.3	25.3
	Explainable ERR, %				-4.9	48.1	35.1	10.3	62.4
50-64	NHW (reference)	3246	1800	1.00	1.00	1.00	1.00	1.00	1.00
	NHB v NHW	981	671	1.52 (1.39-1.66)	1.54 (1.41-1.68)	1.26 (1.14-1.38)	1.39 (1.27-1.52)	1.52 (1.39-1.66)	1.19 (1.08-1.31)
	ERR, %			52.2	53.6	25.9	39.2	51.9	19.0
	Explainable ERR, %				-2.7	50.4	24.9	0.8	63.6
65+	NHW (reference)	3873	2577	1.00	1.00	1.00	1.00	1.00	1.00
	NHB v NHW	634	453	1.14 (1.04-1.26)	1.18 (1.07-1.31)	1.04 (0.93-1.15)	1.08 (0.98-1.20)	1.13 (1.02-1.24)	1.01 (0.91-1.12)

Abbreviations: CI, confidence interval; ERR, excess relative risk; HR, hazard ratio; NHB, non-Hispanic black; NHW, non-Hispanic white.

a. Model one: race.

b. Model two: race, plus age at diagnosis.

c. Model three: race, plus socioeconomic factors (insurance type, marital status and neighborhood socioeconomic status).

d. Model four: race, plus tumor characteristics (histological type, grade, tumor size, regional lymph nodes, and molecular subtype).

e. Model five: race, plus metastatic pattern (number and site of distant metastases).

f. Model six: race, plus age at diagnosis, socioeconomic factors, tumor characteristics, and metastatic pattern.

Table S4. % of Women With Stage IV Breast Cancer, by Race/Ethnicity

	18-49			50-64		
	**Total**	**NHW**	**NHB**	**Total**	**NHW**	**NHB**
No. of patients	20.9	16.3	26.7	39.8	38.2	44.5
Triple negative subtype	14.4	12.6	20	12.6	11.2	19.7
Brain metastases	7.3	6.4	8.8	8.2	8.1	8.6
Tumor size >50 mm	31.9	26.8	36.5	30.6	28.3	35.1
Positive lymph nodes	75.5	72.3	79.3	70..9	69.4	74.7
Multi-site metastases	41.9	40	46.7	43.4	42	45.5

Abbreviations: NHB, non-Hispanic black; NHW, non-Hispanic white.
